# Metabolomic profiling in patients undergoing Off-Pump or On-Pump coronary artery bypass surgery

**DOI:** 10.1186/s12872-017-0518-1

**Published:** 2017-04-05

**Authors:** H. Kirov, M. Schwarzer, S. Neugebauer, G. Faerber, M. Diab, T. Doenst

**Affiliations:** 1 Department of Cardiothoracic Surgery, Friedrich Schiller University Jena, University Hospital, Am Klinikum 1, 07747 Jena, Germany; 2 Department of Cardiothoracic Surgery, Cairo University, Cairo, Egypt; 3 Department of Clinical Chemistry and Laboratory Medicine, Friedrich-Schiller-University Jena, University Hospital, Jena, Germany; 4 Integrated Research and Treatment Center, Center for Sepsis Control and Care (CSCC), Jena, Germany

**Keywords:** Metabolomics, Cardiac surgery, Vasopressor

## Abstract

**Background:**

Coronary artery bypass surgery can be performed without (Off-Pump) or with cardiopulmonary bypass (On-Pump). Extracorporeal circulation and cardioplegic arrest may cause alterations in the plasma metabolome. We assessed metabolomic changes in patients undergoing On-Pump or Off-Pump coronary artery bypass surgery.

**Methods:**

We assessed five analyte classes (41 acylcarnitines, 14 amino acids, 92 glycerophospholipids, 15 sphingolipids, sugars, lactate) using a mass-spectrometry-based kit (Biocrates Absolute***IDQ***® p150) in paired arterial and coronary sinus blood obtained from 10 consecutive On-Pump and 10 Off-Pump patients. Cardioplegia for On-Pump was warm blood Calafiore. On-Pump outcomes were corrected for hemodilution through crystalloid priming.

**Results:**

Demographic data were equal in both groups with normal ejection fraction, renal and liver function. Patients received 2.25 ± 0.64 bypass grafts. All postoperative courses were uneventful. Of 164 measured metabolites, only 13 (7.9%) were altered by cardiopulmonary bypass. We found more long-chain acylcarnitines Off-Pump and more short-chain acylcarnitines On-Pump. Glycerophospholipids showed lower concentrations On-Pump and arginine (as the only different amino acid) Off-Pump. Interestingly, plasma arginine (nitric oxide precursor) concentration at the end of surgery correlated inversely with postoperative vasopressor need (*r* = −0.7; *p* < 0.001). Assessing arterial/venous differences revealed phosphatidylcholine-production and acylcarnitine-consumption. These findings were unaffected by cardiopulmonary bypass, cardioplegia or temporary vessel occlusion during Off-Pump surgery.

**Conclusions:**

Cardiopulmonary bypass and warm blood cardioplegia cause only minor changes to the metabolomic profile of patients undergoing coronary artery bypass surgery. The observed changes affected mainly acylcarnitines. In addition, there appears to be a relationship between arginine and vasopressor need after bypass surgery.

**Electronic supplementary material:**

The online version of this article (doi:10.1186/s12872-017-0518-1) contains supplementary material, which is available to authorized users.

## Background

Novel molecular technologies have been applied to assess the impact of metabolic intermediates on outcome in cardiac surgery. Recent studies have shown that specific metabolic profiles may independently predict adverse events after coronary artery bypass grafting (CABG) [1] or in heart failure patients and after left ventricular assist device (LVAD) implantation [2]. While these investigations addressed the metabolic signatures either before and/or after the surgical procedure, the direct impact of cardiopulmonary bypass surgery has thus far not been addressed. However, cardiopulmonary bypass has been implicated with a plethora of alterations and changes in many systems of the human organism (e.g. inflammatory cascades, coagulation system, individual organ function) [3]. It is therefore well conceivable that the use of cardiopulmonary bypass also results in significant alterations in the metabolomic plasma profile.

CABG without cardiopulmonary bypass (Off-Pump) has been one area, where surgeons have tried to avoid the potentially detrimental effects of cardiopulmonary bypass (On-Pump) [4]. However, the controversial benefits and risks of Off-Pump compared to On-Pump CABG are still a subject of an ongoing discussion [5, 6].

We thus hypothesized that patients undergoing CABG On-Pump and those operated Off-Pump would show substantial differences in their plasma metabolomic profile. We tested our hypothesis in a proof-of-principle type study using targeted metabolomic analysis.

## Methods

### Patient population

A total of 20 consecutive patients undergoing CABG were included in the study. The first 10 patients were operated Off-Pump and the next 10 with cardioplegic arrest. The institutional ethic review committee approved the study protocol (reference number: 3194-07/11) and all patients provided written informed consent. Inclusion criteria were age between 30 and 80 years, Body Mass Index < 30, left ventricular ejection fraction between 50 and 80% and planned elective, isolated, coronary bypass surgery. End stage liver and renal insufficiency, ongoing infection, immunosuppressive therapy and tumours were exclusion criteria.

### Study protocol and sample collection

All patients included in this study received standardized preoperative anaesthetic preparation. The technical approach was similar and in all cases, distal anastomoses were performed before the proximal ones. The patients received insulin infusions as required to attain euglycemia.

In the On-Pump group, immediately after cardiopulmonary bypass was established, a retrograde cardioplegia catheter was inserted in the coronary sinus (CS) and used to gather blood samples. Its correct position was verified manually. Antegrade warm blood cardioplegia was used every 20 min. No retrograde cardioplegia was given, as the retrograde catheter was used only for drawing blood. In both groups, paired arterial and CS blood samples were collected simultaneously at baseline (immediately after CS catheter placement and before aortic cross clamping in the On-Pump group) and immediately after each distal anastomosis was completed. Samples were taken slowly to avoid haemolysis and the first 1 ml was discarded to avoid possible contamination with right atrial blood. They were drawn into EDTA- treated tubes (S-Monovette® EDTA K_2_ Gel, Saarstedt, Nuembrecht, Germany). All samples were immediately centrifuged at 4 ° C and 5000 rpm for 10 min and plasma aliquots were stored in liquid nitrogen.

### Analytical rationale

We used the obtained samples to perform the following analyses. First, a comparison of arterial blood samples from on- and off pump surgery in order to obtain information on the influence of cardiopulmonary bypass on the plasma metabolome. Second, we compared arterial and coronary sinus blood samples in oder to obtain information on the transcoronary changes in the metabolome with and without cardiopulmonary bypass. Finally, we attempted to assess the influence of regional (off pump) and global ischemia (on pump with cardioplegia) with respect to changes in the plasma metabolome.

### Plasma metabolite analyses

Determination of laboratory parameters glucose and lactate was performed using routine diagnostic procedures at an Abbott Architect analyzer (Abbott GmbH, Ludwigshafen, Germany). Glucose was determined with a hexokinase method according to the manufacturer’s recommendations. A colorimetric assay was used for measurement of lactate concentrations.

Metabolite concentrations of five analytic classes (Additional file 1: Table S1) - 41 acylcarnitines, 14 amino acids, 92 glycerophospholipids, 15 sphingolipids and 1 sugar - were measured after preparation of serum according to the manufacturer’s protocol using the Absolute***IDQ***® p150 kit (Biocrates Life Science AG, Innsbruck, Austria) on an API4000™ LC/MS/MS System (AB SCIEX, USA) equipped with an electrospray ionization source, an Agilent G1367B autosampler, and the Analyst 1.51 software (AB SCIEX, USA). In brief, 10 μl of serum was added onto the center kit plate and was dried using a nitrogen evaporator for 30 min. Subsequently, 20 μl of a 5% solution of phenyl-isothiocyanate (Merck) was added. After incubation of 20 min at room temperature, the plate was dried again using an evaporator for 45 min. The metabolites were extracted using 300 μl of a 5 mM ammonium acetate solution in methanol (Merck, Roth). The extracts were obtained after incubation for 30 min on a shaker (450 rpm) by centrifugation at 100 g for 2 min followed by a dilution step with 600 μl of kit MS running solvent. The plate was measured by flow injection analysis and detection of fragments was performed in multiple reaction monitoring mode. Two subsequent 20 μl injections (one for positive and one for negative mode analysis) were injected directly to the MS at a flow of 30 μl/min with MS running solvent. Concentrations for metabolites were determined using the MetIQ™ software package, which is an integral part of the Absolute***IDQ***® kit. These data were exported for following statistical analysis. For analysis only metabolites which appear in at least 50% of the patients were included. Outcomes in the On-Pump group were corrected for hemodilution through crystalloid priming (assessed by the drop in haematocrit). Heatmaps were created with MetaboAnalyst 3.0 software [7, 8]. Data were normalized for each metabolite (autoscaling method, mean-centered and divided by the standard deviation of each variable). A hierarchical clustering in form of a dendrogram for the metabolites using the Pearson distance and the average algorithm was performed.

### Statistical analysis

Statistical analysis was performed via SPSS Statistics 22 (IBM, USA). Normal distribution of the metabolite concentrations was tested. Depending on the outcome either the student-t-test (normal distribution) or the Mann-Whitney-u-test (no normal distribution) was chosen for determination of statistical significance. Pearson correlation was used to investigate the correlation between two variables. Statistical significance was considered for *p*-values < 0.05.

## Results

Table 1 shows the demographic and laboratory data of the patients. There were no differences between the groups. The majority of patients were around 60 years of age and male. There was mild obesity among the patients and approximately one third were diabetic. Ejection fraction was normal in both groups. Preoperative laboratory values showed no evidence of renal or liver dysfunction as well as the absence of major lipid disorders.

**Table 1:** Demographic and laboratory characteristics of the study population

	On-Pump (*n* = 10)	Off-Pump (*n* = 10)	*p* value
Age (years)	67.1 ± 7.53	62.8 ± 3.96	0.133
Male sex (%)	80	90	0.531
BMI (kg/m^2^)	27.84 ± 1.95	27.75 ± 2.2	0.920
Diabetes mellitus (%)	40	30	0.660
Left ventricular ejection fraction (%)	59.3 ± 8.43	63.3 ± 8.6	0.309
Creatinine (μmol/l)	75.3 ± 9.47	78.8 ± 7.05	0.361
CRP (mg/l)	4.51 ± 6.19	2.22 ± 4.53	0.358
ASAT (μmol/l*s)	0.53 ± 0.1	0.57 ± 0.34	0.699
ALAT (μmol/l*s)	0.73 ± 0.23	0.734 ± 0.4	0.979
γ-GT (μmol/l*s)	0.94 ± 0.56	0.67 ± 0.37	0.222
Cholesterol (mmol/l)	4.54 ± 1.07	5.06 ± 1.21	0.332
Triglycerides (mmol/l)	3.02 ± 2.37	2.17 ± 1.45	0.350

Values are mean ± standard deviation or percent of patients

*BMI* body mass index, *CRP* C-reactive protein, *ASAT* aspartate aminotransferase, *ALAT* alanine transaminase, *γ-GT* γ-Glutamyltransferase

Table 2 shows laboratory values and preoperative blood-gas analyses. There were no relevant differences in acid-base homeostasis, glucose, lactate or electrolyte concentrations.

**Table 2:** Preoperative laboratory values and arterial blood gas analyses

	On-Pump (*n* = 10)	Off-Pump (*n* = 10)	*p* value
Standard bicarbonate (mmol/l)	25.04 ± 1.06	24.93 ± 1.91	0.891
Base excess (mmol/l)	0.55 ± 1.85	0.43 ± 2.18	0.896
pH	7.42 ± 0.28	7.41 ± 0.53	0.821
pCO_2_ (kPa)	5.06 ± 0.18	5.13 ± 0.64	0.733
pO_2_ (kPa)	15.77 ± 12.56	10.38 ± 2.3	0.213
O_2_ Saturation,	97.24 ± 1.73	95.7 ± 1.96	0.084
Hb (mmol/l)	8.55 ± 1.13	8.16 ± 1.78	0.461
Na ^+^ (mmol/l)	137.88 ± 3.25	136.4 ± 3.65	0.364
K ^+^ (mmol/l)	4.01 ± 0.32	4.03 ± 0.5	0.924
Ca ^2+^ (mmol/l)	1.20 ± 0.2	1.18 ± 0.24	0.131
Glucose (mmol/l)	6.35 ± 1.17	6.65 ± 2.31	0.719
Lactate (mmol/l)	1.29 ± 0.46	1.29 ± 0.45	1.00

Values are mean ± standard deviation

*pCO*_*2*_ carbon dioxide partial pressure, *pO*_*2*_ arterial oxygen partial pressure, *O*_*2*_ *Saturation* arterial oxygen saturation, *Hb* arterial haemoglobin

Table 3 shows the operative characteristics of the study population. The majority of patients received two or three bypass grafts. All had uneventful operations and were successfully discharged. There was no cardiopulmonary bypass time in the Off-Pump group. The duration of the operative procedure was 50 min longer On-Pump.

**Table 3:** Operative characteristics of the study population

	On-Pump (*n* = 10)	Off-Pump (*n* = 10)	*p* value
Single bypass (n)	1	1	
Double bypass (n)	5	6	
Triple bypass (n)	4	3	
Bypass, mean (n)	2.3 ± 0.68	2.2 ± 0.63	0.736
Cardiopulmonary bypass time (min)	92.7 ± 13.81	0	
Aortic cross clamping time (min.)	57.8 ± 14.05	0	
Operating time (min.)	205.6 ± 13.09	154.2 ± 35.7	0.001

Values are presented as mean ± standard deviation or number of patients

**Fig. 1:**
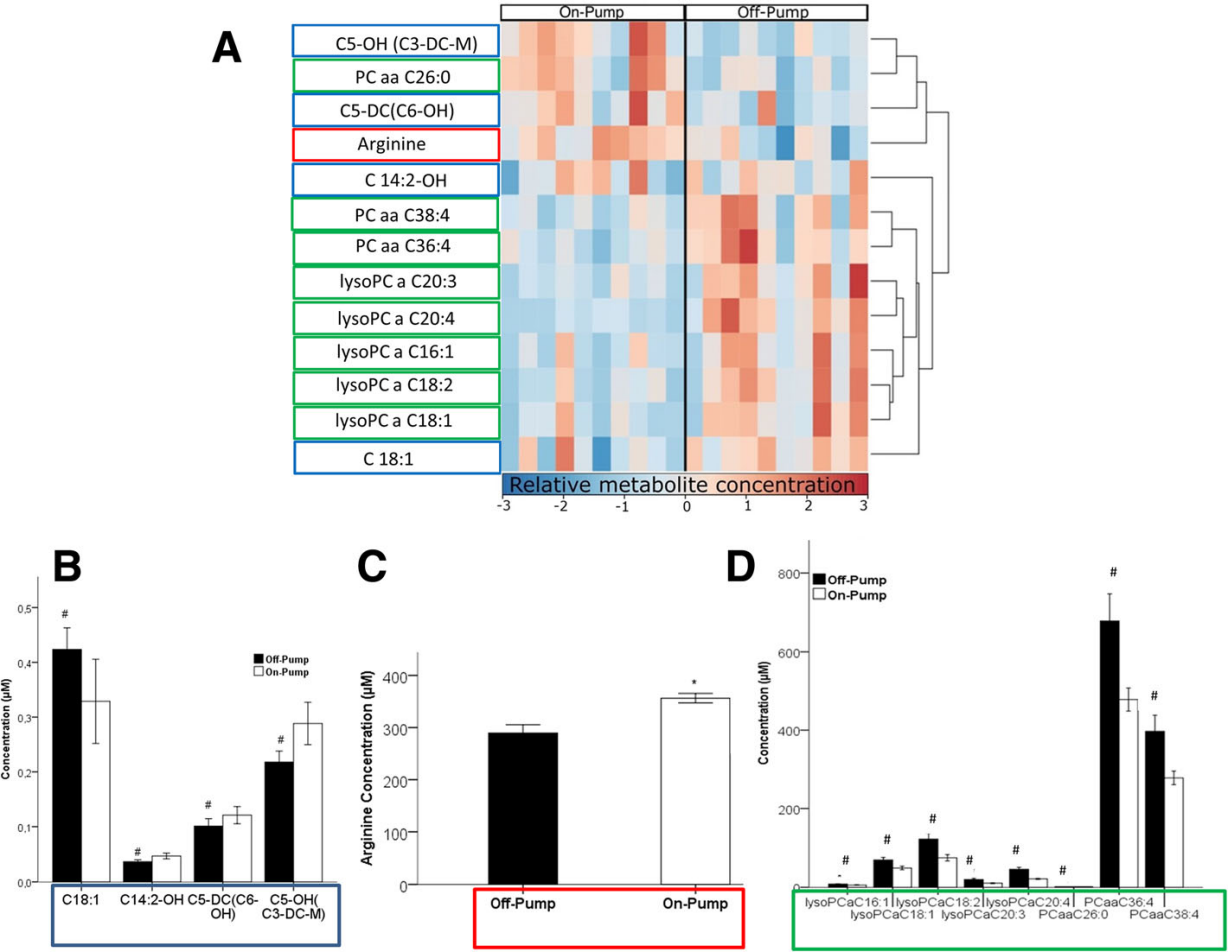
Heatmap (**a**) showing a graphic representation of the differences in metabolite concentrations in the Off-Pump and On-Pump groups, measured at the beginning of the operation. Rows represent the metabolites and columns the samples. The Pearson distance and the average algorithm were used for the dendrogram for the metabolites on the right hand side. Panels **b**, **c** and **d** represent a quantitative comparison in form of a bar diagram of acylcarnitines (**b**), arginine (**c**) and glycerophospholipids (**d**) concentrations compared to On-Pump. # and *: *p* < 0.05. Data are presented as mean ± SD. a, acyl; aa, acyl-acyl; Cx:y, where x is the number of carbons in the fatty acid side chain; y is the number of double bonds in the fatty acid side chain; DC, decarboxyl; M, methyl; OH, hydroxyl; PC, phosphatidylcholine

Figure 1 shows the metabolomic comparison of On-Pump and Off-Pump arterial blood samples, drawn at the beginning of the operation. Of the 164 measured metabolites, only 7.9% (*n* = 13) were altered by the establishment of cardiopulmonary bypass (CPB). These differences are presented in the figure in form of a heatmap (Fig. 1a) – and by quantitative comparison of acylcarnitines (Panel B), arginine (Panel C) and glycerophospholipids (Panel D). The heatmap shows significant differences in the colour patterns. More long-chain acylcarnitines (C18:1) were present in the Off-Pump group and more short chain acylcarnitines (C5-DC (C6-OH) and C5-OH (C3-DC-M)) were present in the On-Pump group (Fig. 1b). C14:2 –OH was the only long-chain acylcarnitine that was elevated On-Pump, but here the concentration differences were minor. From the 14 measured amino acids, only arginine was significantly different. Its concentration was higher On-Pump and lower Off-Pump (Fig. 1c). Glycerophospholipid concentrations were higher Off-Pump and lower On-Pump (Fig. 1d).

**Fig. 2:**
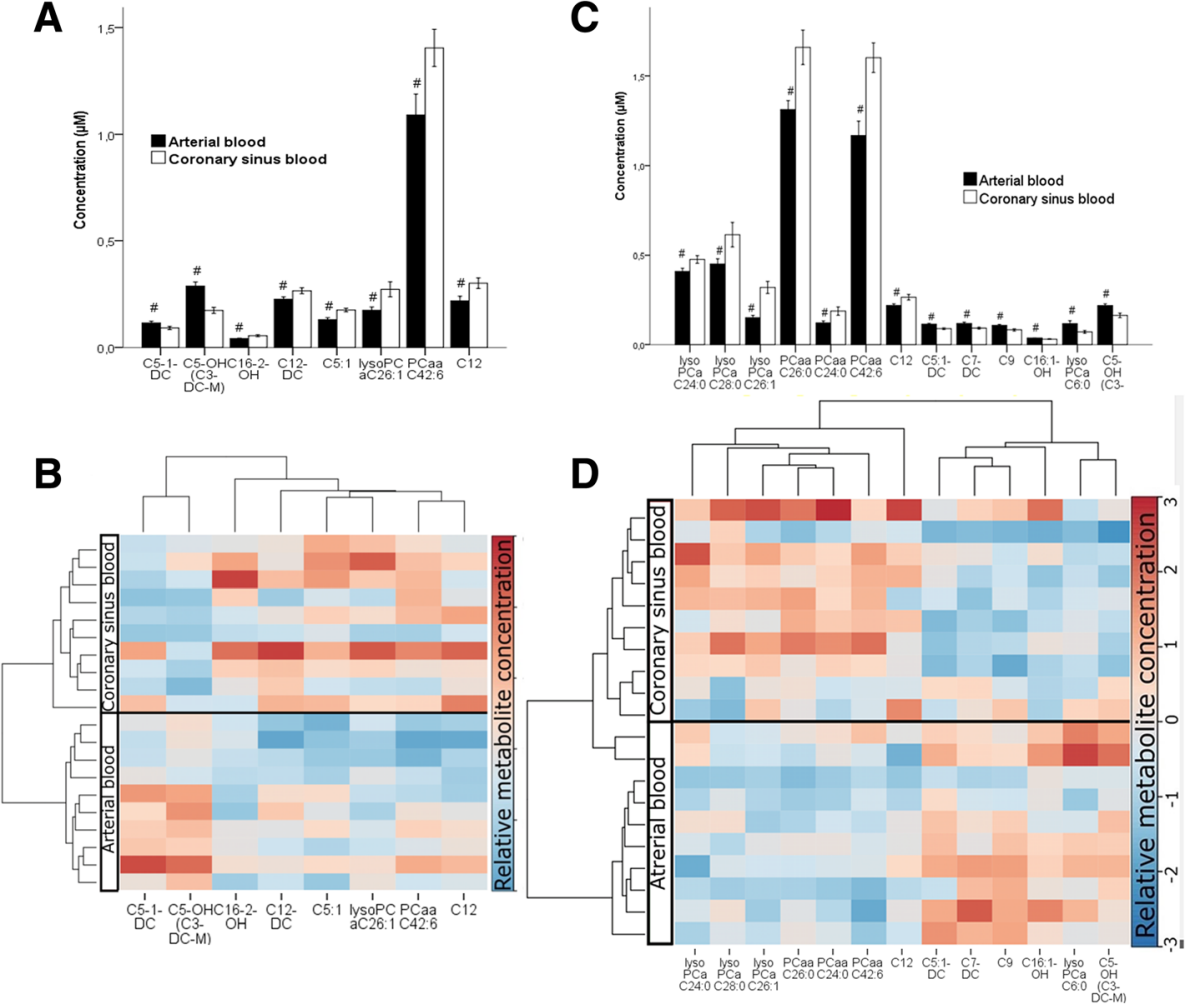
Arterio-venous (coronary sinus) difference in plasma metabolomite composition measured at the beginning of the operation. Panels **a** and **c** represent the quantitative comparison of metabolites in form of a bar diagram for the corresponding heatmap (Panels **b** and **d**). Heatmap rows represent the samples and columns the metabolites. The Pearson distance and the average algorithm were used for the dendrogram for the metabolites on the top. Thus, Panels **a** and **b** represent the On-Pump group and Panels **c** and **d** represent the Off-Pump group. #: *p* < 0.05. Bar diagram data are presented as mean ± SD. a, acyl; aa, acyl-acyl; Cx:y, where x is the number of carbons in the fatty acid side chain; y is the number of double bonds in the fatty acid side chain; DC, decarboxyl; M, methyl; OH, hydroxyl; PC, phosphatidylcholine

Figure 2 shows the arterio-venous (coronary sinus) difference measured at the beginning of the operation. Panel A (quantitative comparison) and Panel B (heatmap) display this differences in the On-Pump group (with established CPB). Panel C (quantitative comparison) and Panel D (heatmap) represent the arterial to coronary sinus difference in the Off-Pump group. On-Pump, during CPB, the heart produced mainly phosphatidylcholines (PCaaC42:6), lysophosphatidylcholines (lysoPCaC26:1) and acylcarnitines (C5:1, C12, C12-DC, C16-2-OH) and consumed only acylcarnitines (C5-1-DC, C5-OH). In general, the changes were similar in the Off-Pump patients with production of mainly phosphatidylcholines (PCaaC26:0, PCaaC24:0, PCaaC42:6), lysophosphatidylcholines (lysoPCaC24:0, lysoPCaC28:0, lysoPCaC26:1) acylcarnitines (C12) and consumption of acylcarnitines (C5:1-DC, C7-DC, C9, C16:1-OH, C5-OH) and only one lysophosphatidylcholine- lysoPcaC6:0.

**Fig. 3:**
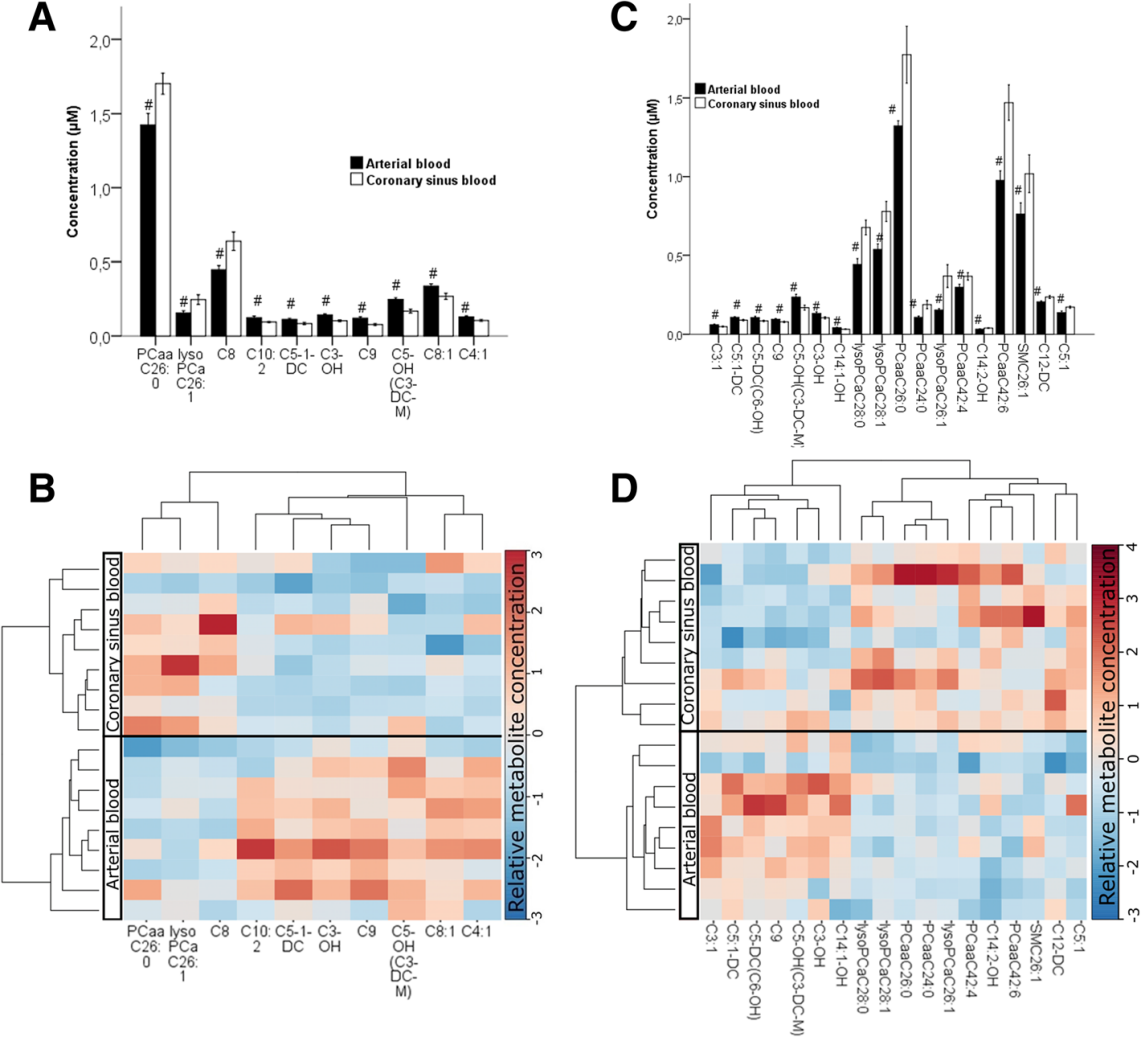
Arterio-venous (coronary sinus) difference in plasma metabolomite composition measured at the end of the second distal anastomosis. Panels **a** and **c** represent the quantitative comparison of metabolites in form of a bar diagram for the corresponding heatmap (Panels **b** and **d**). Heatmap rows represent the samples and columns the metabolites. The Pearson distance and the average algorithm were used for the dendrogram for the metabolites on the top. Thus, Panels **a** and **c** represent the On-Pump group and Panels **b** and **d** represent the Off-Pump group. #: *p* < 0.05. Bar diagram data are presented as mean ± SD. a, acyl; aa, acyl-acyl; Cx:y, where x is the number of carbons in the fatty acid side chain; y is the number of double bonds in the fatty acid side chain; DC, decarboxyl; M, methyl; OH, hydroxyl; PC, phosphatidylcholine; SM, sphingomyelin

Figure 3 illustrates the arterio-coronary sinus differences, similar to Fig. 2, but from samples taken from the end of the second anastomosis- i.e. after an expected ischemic event. Again, panels A and B represent the On-Pump group and panels C and D the Off-Pump group. The already observed trends in the arterial to coronary sinus difference remained similar and were not affected by blood cardioplegia or by temporary vessel occlusion during Off-Pump surgery.

**Fig. 4:**
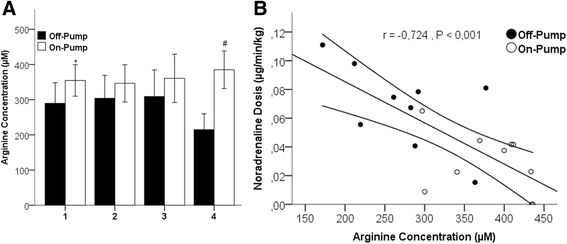
**a** Comparison of mean arterial arginine concentration measured at the beginning of the operation (1), after the first (2), second (3) and third (4) distal anastomosis in the On-Pump (*white*) and Off-Pump (*black*) group.* *p* < 0.001 and # *p* < 0.05. Data are presented as mean ± SEM. **b** Pearson correlation between postoperative noradrenaline doses and arterial arginine concentration measured at the time of the last distal anastomosis in the On-Pump (○) and Off-Pump (●) group including regression line and 95% confidence interval

Figure 4 shows a comparison between the arterial plasma arginine concentrations in the Off-Pump and On-Pump groups at all measured time points (panel A) as well as the correlation between arterial arginine concentration and vasopressor need (Panel B). The Off-Pump patients had lower arginine concentrations. This difference increased with time and was greatest at the last measured time point (A). This difference in concentration was related to postoperative vasopressor requirements (B). There was a strong correlation between the noradrenaline concentration administered at the end of the operation and the last measured intraoperative arginine value. Thus, the On-Pump patients had high arginine concentrations and low postoperative noradrenaline doses and, vice versa, Off-Pump patients had low arginine concentrations but higher postoperative noradrenaline requirements.

## Discussion

We demonstrate in this manuscript that cardiopulmonary bypass and warm blood cardioplegia cause only minor changes to the metabolomic profile of patients undergoing bypass surgery and that acylcarnitines are elevated in both Off-Pump and On-Pump, but chain lengths differ. Furthermore, we demonstrate that there appears to be a correlation between arterial arginine concentration and vasopressor need after bypass surgery.

One would expect that establishing cardiopulmonary bypass leads to major changes in the metabolomic composition of the blood. However, the overall changes we observed were rather minor. Less than 10% of metabolites were altered (Fig. 1). This is striking because investigations addressing the activation of signalling cascades through cardiopulmonary bypass show substantial derangements [9, 10]. For instance, inflammatory processes have been suggested to be activated by the exposure of blood to external surfaces [10, 11]. Since inflammatory cascades directly influence the expression of genes and the function of the cell [12], it would be no surprise to see measureable changes in metabolites associated with such activations. Some of those genes for example are relevant for the facilitation of glucose transport in tissues with a high glucose demand (Solute carrier family 2, member 3) or gluconeogenesis (Phosphoenolpyruvate carboxykinase 2) [13]. That would mean that we could expect changes in the levels of glucose or the metabolites connected with glycolysis and gluconeogenesis. However, the levels of glucose and lactate were not different between On-Pump and Off-pump in our analysis. Thus, it may be one conclusion that the use of cardiopulmonary bypass is less relevant than the type of surgery (i.e. bypass surgery) or the surgical approach. Irrespective of the magnitude of the overall changes, we still identified several changes in individual metabolite classes that may be relevant for long or short-term outcome and therefore require further discussion.

We demonstrate that patients on cardiopulmonary bypass have significantly elevated levels of short-chain acylcarnitines and lower levels of long-chain acylcarnitines such as C18:1 compared to Off-Pump patients (Fig. 1b). Plasma acylcarnitines are products of incomplete β-oxidation and might be elevated due to inborn or other errors of mitochondrial fatty acid oxidation. Short- and medium-chain acylcarnitines are significantly increased in acute myocardial infarction and chest pain patients whereas the levels of long-chain acylcarnitines such as C18:1 and C18:2 are insignificantly decreased in these patients [14]. Short-chain acylcarnitines are also known to be predictive of myocardial infarction, repeat revascularisation or death at any time following CABG [1]. Applying these data to our results may suggest that On-Pump patients are possibly at higher risk of myocardial infarction, repeat revascularization or death, compared to Off-Pump patients. However, in heart failure patients, long-chain acylcarnitines have been associated with poor long term outcomes [2]. Thus, it appears that chain length may not be the most important part of this association. In addition, these studies assessed the role of acylcarnitines before surgery and/or during follow-up and did not address the changes during surgery as we did. Since current evidence does not support any major differences in long term outcome between On- and Off-Pump CABG, it is unlikely that the observed metabolomic alterations during surgery may have substantial impact.

We also demonstrate a significant correlation between arterial arginine concentration at the end of surgery and postoperative vasopressor needs in the immediately following time period in the intensive care unit (Fig. 4). Off-Pump patients had lower arginine concentrations and required more vasopressor therapy. In contrast, On-Pump patients had higher arginine concentrations but lower vasopressor needs. Arginine is the primary substrate for the synthesis of nitric oxide in the human body. Nitric oxide is produced from arginine through the enzyme nitric oxide synthase, which has three isoforms: endothelial (eNOS), neuronal (nNOS), and inducible (iNOS). Arginine deficiency is suggested to be the result of decreased arginine uptake or an impaired arginine de novo synthesis from citrulline [15]. The latter may appear in combination with an enhanced arginine catabolism by the upregulation of arginase and the inflammatory nitric oxide synthase (iNOS; NOS2) in the immune response [15]. It is also known that activated through the immune response, macrophages actively import arginine to synthesize NO by NOS2 [16, 17]. However, the NOS2 in the macrophages can also be inhibited through interleukins, such as IL-10 [18, 19]. We did not measure IL-10 in this study but this has been done before by others in the past [20–22]. Several authors have shown higher IL-10 levels On-Pump than Off-Pump at the end of surgery and early postoperatively [20–22]. Assuming that same is true in our patients, it is conceivable that IL-10 at the end of the operation inhibits iNOS in On-Pump CABG, resulting in less NO production and decreased arginine catabolism through iNOS. The consequence is higher arginine in plasma and less vascular dilatation requiring less vasopression from noradrenaline. Irrespective of the above described potential mechanism, it is not clear whether arginine is truly involved in the mechanism leading to vasopressor requirements. However, the significant correlation shown in Fig. 4 is striking and requires further investigation because it may reveal new understanding for the postoperative vasopressor management of patients undergoing cardiac surgery On- or Off-Pump.

## Conclusions

In conclusion, we demonstrate in this study that cardiopulmonary bypass and warm blood cardioplegia cause only minor changes to the metabolomic profile of patients undergoing bypass surgery and that acylcarnitines are elevated in both Off-Pump and On-Pump cases, but chain lengths differ. Furthermore, we demonstrate that there appears to be a correlation between arterial arginine concentration and vasopressor need after bypass surgery.

## Additional file

Additional file 1:
**Table S1.** List of metabolites used in the analysis and corresponding abbreviations. (DOCX 27 kb)
