# Supplementary material for: Metabolomic profiling in patients undergoing Off-Pump or On-Pump coronary artery bypass surgery
Source: BMC Cardiovasc Disord. 2017 Apr 5;17:93. doi: 10.1186/s12872-017-0518-1 (PMC5381030; doi:10.1186/s12872-017-0518-1)
Supplement: Additional file 1: — Table S1. List of metabolites used in the analysis and corresponding abbreviations. (DOCX 27 kb) [file 12872_2017_518_MOESM1_ESM.docx]

Additional file 1: Table S1.

| Number | MetIQ Short Name | Biochemical Name | | | |
| --- | --- | --- | --- | --- | --- |
| 1 | C0 | DL-Carnitine | | |  |
| 2 | C2 | Acetyl-L-carnitine | | |  |
| 3 | C3 | Propionyl-L-carnitine | | |  |
| 4 | C3:1 | Propenoyl-L-carnitine | | |  |
| 5 | C3-OH | Hydroxypropionyl-L-carnitine | | |  |
| 6 | C4 | Butyryl-L-carnitine | | |  |
| 7 | C4:1 | Butenyl-L-carnitine | | |  |
| 8 | C4-OH | Hydroxybutyryl-L-carnitine | | |  |
| 9 | C5 | Valeryl-L-carnitine | | |  |
| 10 | C5:1 | Tiglyl-L-carnitine | | |  |
| 11 | C5:1-DC | Glutaconyl-L-carnitine | | |  |
| 12 | C5-DC (C6-OH) | Glutaryl-L-carnitine (Hydroxyhexanoyl-L-carnitine) | | |  |
| 13 | C5-M-DC | Methylglutaryl-L-carnitine | | |  |
| 14 | C5-OH (C3-DC-M) | Hydroxyvaleryl-L-carnitine (Methylmalonyl-L-carnitine) | | |  |
| 15 | C6 (C4:1-DC) | Hexanoyl-L-carnitine (Fumaryl-L-carnitine) | | |  |
| 16 | C6:1 | Hexenoyl-L-carnitine | | |  |
| 17 | C7-DC | Pimelyl-L-carnitine | | |  |
| 18 | C8 | Octanoyl-L-carnitine | | |  |
| 19 | C8:1 | Octenoyl-L-carnitine | | |  |
| 20 | C9 | Nonayl-L-carnitine | | |  |
| 21 | C10 | Decanoyl-L-carnitine | | |  |
| 22 | C10:1 | Decenoyl-L-carnitine | | |  |
| 23 | C10:2 | Decadienyl-L-carnitine | | |  |
| 24 | C12 | Dodecanoyl-L-carnitine | | |  |
| 25 | C12:1 | Dodecenoyl-L-carnitine | | |  |
| 26 | C14 | Tetradecanoyl-L-carnitine | | |  |
| 27 | C14:1 | Tetradecenoyl-L-carnitine | | |  |
| 28 | C14:1-OH | Hydroxytetradecenoyl-L- carnitine | | |  |
| 29 | C14:2 | Tetradecadienyl-L-carnitine | | |  |
| 30 | C14:2-OH | Hydroxytetradecadienyl-L- carnitine | | |  |
| 31 | C16 | Hexadecanoyl-L-carnitine | | |  |
| 32 | C16:1 | Hexadecenoyl-L-carnitine | | |  |
| 33 | C16:1-OH | Hydroxyhexadecenoyl-L- carnitine | | |  |
| 34 | C16:2 | Hexadecadienyl-L-carnitine | | |  |
| 35 | C16:2-OH | Hydroxyhexadecadienyl-L- carnitine | | |  |
| 36 | C16-OH | Hydroxyhexadecanoyl-L- carnitine | | |  |
| 37 | C18 | Octadecanoyl-L-carnitine | | |  |
| 38 | C18:1 | Octadecenoyl-L-carnitine | | |  |
| 39 | C18:1-OH | Hydroxyoctadecenoyl-L- carnitine | | |  |
| 40 | C18:2 | Octadecadienyl-L-carnitine | | |  |
| 41 | Arg | Arginine | | |  |
| 42 | Gln | Glutamine | | |  |
| 43 | Gly | Glycine | | |  |
| 44 | His | Histidine | | |  |
| 45 | Met | Methionine | | |  |
| 46 | Orn | Ornithine | | |  |
| 47 | Phe | Phenylalanine | | |  |
| 48 | Pro | Proline | | |  |
| 49 | Ser | Serine | | |  |
| 50 | Thr | Threonine | | |  |
| 51 | Trp | Tryptophan | | |  |
| 52 | Tyr | Tyrosine | | |  |
| 53 | Val | Valine | | |  |
| 54 | xLeu | xLeucine | | |  |
| 55 | H1 | Hexose | | |  |
| 56 | lysoPC a C6:0 | lysoPhosphatidylcholine acyl C6:0 | | |  |
| 57 | lysoPC a C14:0 | lysoPhosphatidylcholine acyl C14:0 | | |  |
| 58 | lysoPC a C16:0 | lysoPhosphatidylcholine acyl C16:0 | | |  |
| 59 | lysoPC a C16:1 | lysoPhosphatidylcholine acyl C16:1 | | |  |
| 60 | lysoPC a C17:0 | lysoPhosphatidylcholine acyl C17:0 | | |  |
| 61 | lysoPC a C18:0 | lysoPhosphatidylcholine acyl C18:0 | | |  |
| 62 | lysoPC a C18:1 | lysoPhosphatidylcholine acyl C18:1 | | |  |
| 63 | lysoPC a C18:2 | lysoPhosphatidylcholine acyl C18:2 | | |  |
| 64 | lysoPC a C20:3 | lysoPhosphatidylcholine acyl C20:3 | | |  |
| 65 | lysoPC a C20:4 | lysoPhosphatidylcholine acyl C20:4 | | |  |
| 66 | lysoPC a C24:0 | lysoPhosphatidylcholine acyl C24:0 | | |  |
| 67 | lysoPC a C26:0 | lysoPhosphatidylcholine acyl C26:0 | | |  |
| 68 | lysoPC a C26:1 | lysoPhosphatidylcholine acyl C26:1 | | |  |
| 69 | lysoPC a C28:0 | lysoPhosphatidylcholine acyl C28:0 | | |  |
| 70 | lysoPC a C28:1 | lysoPhosphatidylcholine acyl C28:1 | | |  |
| 71 | PC aa C24:0 | Phosphatidylcholine diacyl C24:0 | | |  |
| 72 | PC aa C26:0 | Phosphatidylcholine diacyl C26:0 | | |  |
| 73 | PC aa C28:1 | Phosphatidylcholine diacyl C28:1 | | |  |
| 74 | PC aa C30:0 | Phosphatidylcholine diacyl C30:0 | | |  |
| 75 | PC aa C30:2 | Phosphatidylcholine diacyl C30:2 | | |  |
| 76 | PC aa C32:0 | Phosphatidylcholine diacyl C32:0 | | |  |
| 77 | PC aa C32:1 | Phosphatidylcholine diacyl C32:1 | | |  |
| 78 | PC aa C32:2 | Phosphatidylcholine diacyl C32:2 | | |  |
| 79 | PC aa C32:3 | Phosphatidylcholine diacyl C32:3 | | |  |
| 80 | PC aa C34:1 | Phosphatidylcholine diacyl C34:1 | | |  |
| 81 | PC aa C34:2 | Phosphatidylcholine diacyl C34:2 | | |  |
| 82 | PC aa C34:3 | Phosphatidylcholine diacyl C34:3 | | |  |
| 83 | PC aa C34:4 | Phosphatidylcholine diacyl C34:4 | | |  |
| 84 | PC aa C36:0 | Phosphatidylcholine diacyl C36:0 | | |  |
| 85 | PC aa C36:1 | Phosphatidylcholine diacyl C36:1 | | |  |
| 86 | PC aa C36:2 | Phosphatidylcholine diacyl C36:2 | | |  |
| 87 | PC aa C36:3 | Phosphatidylcholine diacyl C36:3 | | |  |
| 88 | PC aa C36:4 | Phosphatidylcholine diacyl C36:4 | | |  |
| 89 | PC aa C36:5 | Phosphatidylcholine diacyl C36:5 | | |  |
| 90 | PC aa C36:6 | Phosphatidylcholine diacyl C36:6 | | |  |
| 91 | PC aa C38:0 | Phosphatidylcholine diacyl C38:0 | | |  |
| 92 | PC aa C38:1 | Phosphatidylcholine diacyl C38:1 | | |  |
| 93 | PC aa C38:3 | Phosphatidylcholine diacyl C38:3 | | |  |
| 94 | PC aa C38:4 | Phosphatidylcholine diacyl C38:4 | | |  |
| 95 | PC aa C38:5 | Phosphatidylcholine diacyl C38:5 | | |  |
| 96 | PC aa C38:6 | Phosphatidylcholine diacyl C38:6 | | |  |
| 97 | PC aa C40:1 | Phosphatidylcholine diacyl C40:1 | | |  |
| 98 | PC aa C40:2 | Phosphatidylcholine diacyl C40:2 | | |  |
| 99 | PC aa C40:3 | Phosphatidylcholine diacyl C40:3 | | |  |
| 100 | PC aa C40:4 | Phosphatidylcholine diacyl C40:4 | | |  |
| 101 | PC aa C40:5 | Phosphatidylcholine diacyl C40:5 | | |  |
| 102 | PC aa C40:6 | Phosphatidylcholine diacyl C40:6 | | |  |
| 103 | PC aa C42:0 | Phosphatidylcholine diacyl C42:0 | | |  |
| 104 | PC aa C42:1 | Phosphatidylcholine diacyl C42:1 | | |  |
| 105 | PC aa C42:2 | Phosphatidylcholine diacyl C42:2 | | |  |
| 106 | PC aa C42:4 | Phosphatidylcholine diacyl C42:4 | | |  |
| 107 | PC aa C42:5 | Phosphatidylcholine diacyl C42:5 | | |  |
| 108 | PC aa C42:6 | Phosphatidylcholine diacyl C42:6 | | |  |
| 109 | PC ae C30:0 | Phosphatidylcholine acyl-alkyl C30:0 | | |  |
| 110 | PC ae C30:1 | Phosphatidylcholine acyl-alkyl C30:1 | | |  |
| 111 | PC ae C30:2 | Phosphatidylcholine acyl-alkyl C30:2 | | |  |
| 112 | PC ae C32:1 | Phosphatidylcholine acyl-alkyl C32:1 | | |  |
| 113 | PC ae C32:2 | Phosphatidylcholine acyl-alkyl C32:2 | | |  |
| 114 | PC ae C34:0 | Phosphatidylcholine acyl-alkyl C34:0 | | |  |
| 115 | PC ae C34:1 | Phosphatidylcholine acyl-alkyl C34:1 | | |  |
| 116 | PC ae C34:2 | Phosphatidylcholine acyl-alkyl C34:2 | | |  |
| 117 | PC ae C34:3 | Phosphatidylcholine acyl-alkyl C34:3 | | |  |
| 118 | PC ae C36:0 | Phosphatidylcholine acyl-alkyl C36:0 | | |  |
| 119 | PC ae C36:1 | Phosphatidylcholine acyl-alkyl C36:1 | | |  |
| 120 | PC ae C36:2 | Phosphatidylcholine acyl-alkyl C36:2 | | |  |
| 121 | PC ae C36:3 | Phosphatidylcholine acyl-alkyl C36:3 | | |  |
| 122 | PC ae C36:4 | Phosphatidylcholine acyl-alkyl C36:4 | | |  |
| 123 | PC ae C36:5 | Phosphatidylcholine acyl-alkyl C36:5 | | |  |
| 124 | PC ae C38:0 | Phosphatidylcholine acyl-alkyl C38:0 | | |  |
| 125 | PC ae C38:1 | Phosphatidylcholine acyl-alkyl C38:1 | | |  |
| 126 | PC ae C38:2 | Phosphatidylcholine acyl-alkyl C38:2 | | |  |
| 127 | PC ae C38:3 | Phosphatidylcholine acyl-alkyl C38:3 | | |  |
| 128 | PC ae C38:4 | Phosphatidylcholine acyl-alkyl C38:4 | | |  |
| 129 | PC ae C38:5 | Phosphatidylcholine acyl-alkyl C38:5 | | |  |
| 130 | PC ae C38:6 | Phosphatidylcholine acyl-alkyl C38:6 | | |  |
| 131 | PC ae C40:0 | Phosphatidylcholine acyl-alkyl C40:0 | | |  |
| 132 | PC ae C40:1 | Phosphatidylcholine acyl-alkyl C40:1 | | |  |
| 133 | PC ae C40:2 | Phosphatidylcholine acyl-alkyl C40:2 | | |  |
| 134 | PC ae C40:3 | Phosphatidylcholine acyl-alkyl C40:3 | | |  |
| 135 | PC ae C40:4 | Phosphatidylcholine acyl-alkyl C40:4 | | |  |
| 136 | PC ae C40:5 | Phosphatidylcholine acyl-alkyl C40:5 | | |  |
| 137 | PC ae C40:6 | Phosphatidylcholine acyl-alkyl C40:6 | | |  |
| 138 | PC ae C42:0 | Phosphatidylcholine acyl-alkyl C42:0 | | |  |
| 139 | PC ae C42:1 | Phosphatidylcholine acyl-alkyl C42:1 | | |  |
| 140 | PC ae C42:2 | Phosphatidylcholine acyl-alkyl C42:2 | | |  |
| 141 | PC ae C42:3 | Phosphatidylcholine acyl-alkyl C42:3 | | |  |
| 142 | PC ae C42:4 | Phosphatidylcholine acyl-alkyl C42:4 | | |  |
| 143 | PC ae C42:5 | Phosphatidylcholine acyl-alkyl C42:5 | | |  |
| 144 | PC ae C44:3 | Phosphatidylcholine acyl-alkyl C44:3 | | |  |
| 145 | PC ae C44:4 | Phosphatidylcholine acyl-alkyl C44:4 | | |  |
| 146 | PC ae C44:5 | Phosphatidylcholine acyl-alkyl C44:5 | | |  |
| 147 | PC ae C44:6 | Phosphatidylcholine acyl-alkyl C44:6 | | |  |
| 148 | SM (OH) C14:1 | Hydroxysphingomyeline C14:1 | | |  |
| 149 | SM C16:0 | Sphingomyeline C16:0 | | |  |
| 150 | SM C16:1 | Sphingomyeline C16:1 | | |  |
| 151 | SM (OH) C16:1 | Hydroxysphingomyeline C16:1 | | |  |
| 152 | SM C18:0 | Sphingomyeline C18:0 | | |  |
| 153 | SM C18:1 | Sphingomyeline C18:1 | | |  |
| 154 | SM C20:2 | Sphingomyeline C20:2 | | |  |
| 155 | SM C22:3 | Sphingomyeline C22:3 | | |  |
| 156 | SM (OH) C22:1 | Hydroxysphingomyeline C22:1 | | |  |
| 157 | SM (OH) C22:2 | Hydroxysphingomyeline C22:2 | | |  |
| 158 | SM C24:0 | Sphingomyeline C24:0 | | |  |
| 159 | SM C24:1 | Sphingomyeline C24:1 | | |  |
| 160 | SM (OH) C24:1 | | Hydroxysphingomyeline C24:1 | |  |
| 161 | SM C26:0 | | | Sphingomyeline C26:0 |  |
| 162 | SM C26:1 | | | Sphingomyeline C26:1 |  |
